# Association of weight change patterns across adulthood with incident asthma: a retrospective cohort study

**DOI:** 10.1038/s41598-022-13555-w

**Published:** 2022-06-13

**Authors:** Wei Zhang, Jie Du, Shaochun Wang, Huihui Ma

**Affiliations:** 1 Department of Respiratory and Critical Care Medicine, Shaanxi Provincial People’s Hospital, Xi’an, China; 2 Department of Medical Examination Center, Shaanxi Provincial People’s Hospital, Xi’an, China

**Keywords:** Diseases, Endocrinology, Health care, Risk factors

## Abstract

This study aimed to investigate the relationship between weight change patterns across adulthood and the risk of incident asthma later in life using data from the National Health and Nutrition Examination Survey (NHANES) 2001–2018. In this retrospective cohort study, asthma was defined by self-report questionnaires, and age at diagnosis was used to estimate the time of asthma onset. Based on BMI at 25 years old (young adulthood) and BMI at 10 years before the survey (middle adulthood), patterns of weight change were divided into five categories including stable normal, non-obese to obese, obese to non-obese, maximum overweight and stable obese. A total of 27,359 participants (female 13,582, 49.6%) were enrolled in this study and during a mean follow-up of 9.8 years, 1035 subjects occurred asthma. After adjusting for age, gender, race, education, family income and smoking status, participants changing from non-obese to obese, stable obese had significantly higher risks of incident asthma than those with normal weight during adulthood (HR1.70, 95% CI 1.35–2.15, P < 0.0001; HR 1.66, 95% CI 1.21–2.19 P = 0.0019, respectively). The findings suggested that maintaining normal weight during adulthood may be important for preventing incident asthma in later life.

## Introduction

Asthma is one of the most common chronic airways inflammatory diseases. The prevalence of asthma increased by 13% from 1990 to 2015 affecting an estimated 358 million people, and an additional 100 million asthma diagnoses are expected by 2025 worldwide^1, 2^. Due to frequent exacerbations, hospitalizations, and management of comorbidities, asthma leads to poorer quality of life for patients and increased health care costs. In 2013, the total annual costs to medically treat asthma patients were estimated at $82 billion in the US^3^. Therefore, to decrease the disease burden, it is necessary to identify modifiable risk factors for the incident and morbidity of this disease.

The incidence of obesity has been increasing in the past few decades, with about 2 billion adults with overweight or obese worldwide^4^. Obesity has been shown to be a risk factor for asthma and there is a U-shaped or J-shaped relationship between a single measurement BMI in adulthood and the risk of asthma according to previous studies^5–7^. In addition, several epidemiological studies found that weight gain during different life stages is also associated with an increased incidence of asthma, including weight gain from birth to childhood and from childhood to young adulthood^8–13^.

Two recent studies used KNHANES and NHANES data to analyze the association between weight change from young adulthood to midlife and incident asthma, the results of the two studies were inconsistent^14, 15^. In addition, there were more overweight participants than obese participants. In the previous study, weight status was divided into obese and non-obese, with overweight included in the non-obese group as a reference group^15^. The relationship between weight changed in overweight participants and the risk of asthma was unclear. Therefore, in order to better explain the relationship between the change of normal weight, overweight, and obesity and the risk of asthma, we used NHANES (2001–2018) to investigate the relationship between weight change and the risk of asthma later.

## Results

### Baseline characteristics of the participants and weight change pattern

**Figure 1:**
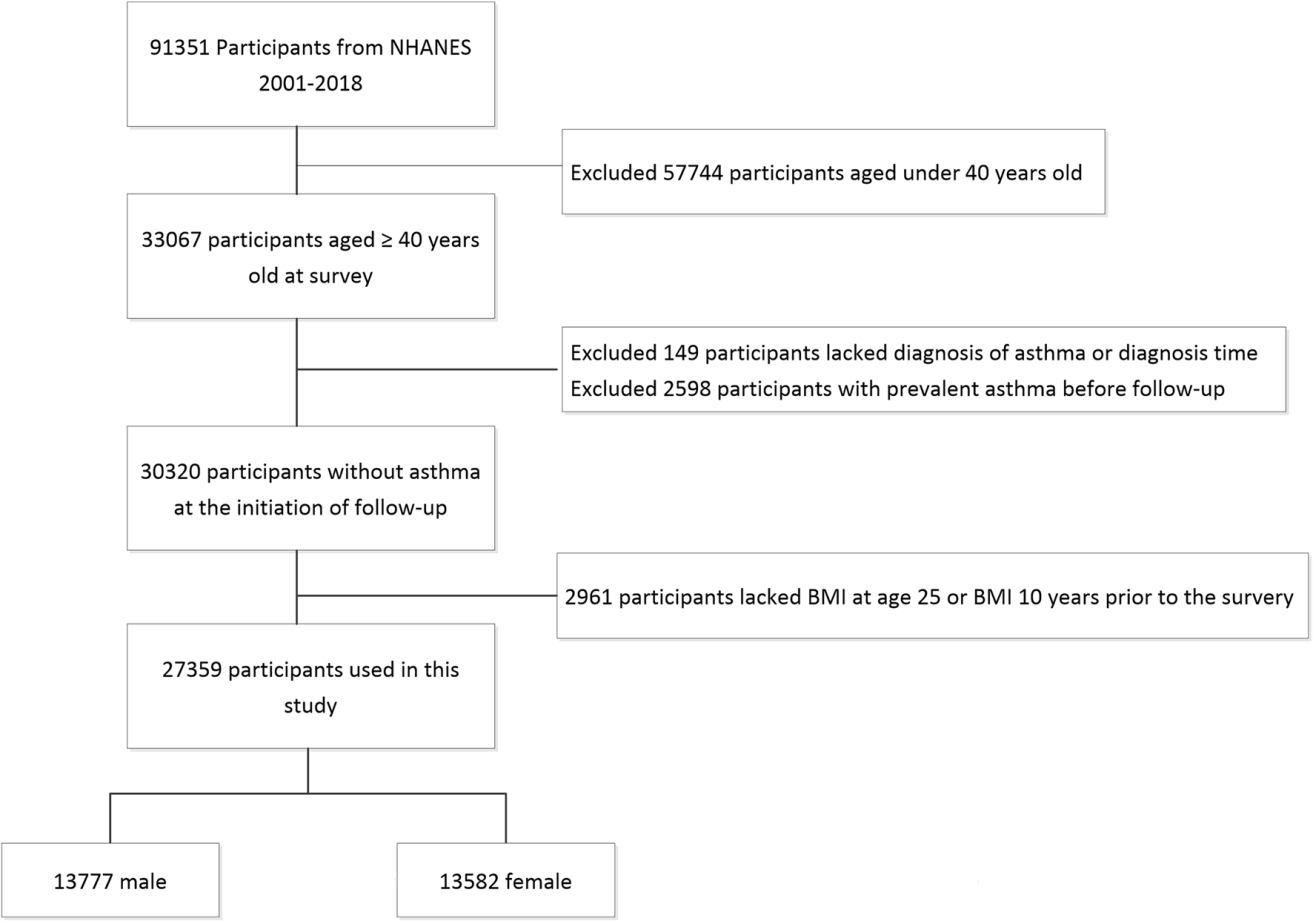
Study inclusion/exclusion criteria for analytic sample, NHANES 2001–2018.

The characteristics of the study population according to weight change patterns with weighted estimates are shown in Table 1, and the flowchart of the study is presented in Fig. 1. 27,359 participants were included in this study. The mean (SD) age was 50.3 (12.8) years. The mean (SD) BMI of the participants at age 25, 10 years before baseline and baseline was 23.3 (4.4) kg/m^2^, 27.1 (5.9) kg/m^2^, and 28.3 (6.0) kg/m^2^, respectively. 49.6% of participants were female. The range of age at 10 years before baseline was 30–75 years.

**Table 1:** Baseline characteristics of study participants in the NHANES 2001–2018 according to weight change patterns during adulthood.

Characteristics	Weight change patterns				
	Stable normal	Maximum overweight	Obese to non-obese	Non-obese to obese	Stable obese
Participants	10,379 (37.9%)	10,215 (37.3%)	255 (0.9%)	5001 (18.3%)	1509 (5.5%)
Mean (95% CI) age, years	46.4 ± 0.2	48.4 ± 0.2	45.1 ± 0.9	49.8 ± 0.3	42.1 ± 0.3
Female	6151 (59.3%)	4065 (39.8%)	107 (42.0%)	2524 (50.5%)	735 (48.7%)
**Mean (95% CI) body mass index**					
At age 25 years	20.6 ± 0.0	23.6 ± 0.0	33.8 ± 0.4	24.8 ± 0.1	34.5 ± 0.1
At 10 years before baseline	22.2 ± 0.0	27.1 ± 0.0	26.9 ± 0.2	34.0 ± 0.1	38.5 ± 0.3
At baseline	24.1 ± 0.0	28.7 ± 0.1	30.3 ± 0.5	33.6 ± 0.1	37.8 ± 0.3
Absolute weight change, kg	4.1 ± 0.1	9.5 ± 0.1	− 19.2 ± 1.5	25.2 ± 0.3	10.9 ± 0.7
**Race/ethnicity**					
Mexican American	1169 (11.3%)	1593 (15.6%)	50 (19.6%)	812 (16.2%)	277 (18.4%)
Non-Hispanic white	2198 (21.2%)	1552 (15.2%)	40 (15.7%)	591 (11.8%)	156 (10.3%)
Non-Hispanic black	5112 (49.3%)	4988 (48.8%)	110 (43.1%)	2386 (47.7%)	607 (40.2%)
Other race	1900 (18.3%)	2082 (20.4%)	55 (21.6%)	1212 (24.2%)	469 (31.1%)
**Education**					
Less than high school	2516 (24.3%)	2793 (27.4%)	96 (37.6%)	1390 (27.8%)	444 (29.5%)
High school or equivalent	2446 (23.6%)	2461 (24.1%)	52 (20.4%)	1236 (24.7%)	373 (24.8%)
College or above	5407 (52.1%)	4953 (48.5%)	107 (42.0%)	2372 (47.5%)	690 (45.8%)
**Family income-poverty ratio level**					
0–1.0	1573 (15.2%)	1383 (13.5%)	60 (23.5%)	743 (14.9%)	305 (20.2%)
1.1–3.0	3959 (38.1%)	3977 (38.9%)	106 (41.6%)	2079 (41.6%)	589 (39.0%)
> 3.0	3970 (38.3%)	4006 (39.2%)	75 (29.4%)	1749 (35.0%)	487 (32.3%)
**Smoking status**					
Ever smoker	5094 (49.1%)	5134 (50.3%)	144 (56.5%)	2424 (48.5%)	713 (47.3%)
Never smoker	5278 (50.9%)	5075 (49.7%)	111 (43.5%)	2576 (51.5%)	795 (52.7%)

All estimates accounted for complex survey designs and the analyses used the sample weights, stratification and clustering suggested by the CDC.

Twenty-three, 2298, and 15 participants had missing information for baseline education level, family income-poverty ratio, and smoking status respectively.

During adulthood, 37.9% of participants maintained normal weight, 5.5% were stable obese, 18.3% gained weight from non-obese to obese, and only 0.9% lost weight from obese to normal weight. People with a stable normal BMI were more likely to be female, non-Hispanic black, non-Hispanic white and had a higher level of education compared with people with other weight patterns.

### Associations of BMI at age 25 and 10 years before baseline and the risk of asthma

In the NHANES 2001–2018 study 1035 participants developed asthma during an average follow-up period of 9.3 years. We evaluated the association between BMI_age 25,_ BMI_10 prior_ and the risk of asthma, which are presented in Supplementary Tables S2 and S3. We found non-linear relationships between BMI _age 25,_ BMI_10 prior_ and incident asthma (Supplementary Fig. S2). Individuals at age 25 with class 2 obesity had a higher risk of asthma (OR 1.93, 95% CI 1.04–3.57) compared with normal weight. When we used normal weight_10 prior_ as the reference, the OR was 1.31 (95% CI 1.04–1.64) for overweight, 1.63 (95% CI 1.28–2.08) for class 1 obesity, 2.15 (95% CI 1.15–3.05) for class 2 obesity and 1.44 (95% CI 1.01–2.06) for class 3 obesity.

### Associations of weight change patterns and the risk of asthma

By using the stable normal group as reference, the stable obese group and non-obese to obese groups are associated with a higher risk of asthma, with HR (95% CI) of 1.66 (1.21, 2.29) and 1.70 (1.35, 2.15) in model 3, respectively (Table 2). By using the stable obese group as the reference, the stable normal group significantly reduced the risk of asthma with HR (95% CI) of 0.60 (0.44, 0.83) as presented in Table 3. Supplementary Figure S3 presented the adjusted cumulative hazard curves of asthma for each weight change pattern, and weight change from non-obesity to obesity had the highest cumulative incidence.

**Table 2:** Hazard ratios (HRs) and 95% confidence intervals (CIs) of incident asthma with weight change patterns across adulthood in NHANES 2001–2018 used the stable normal category as a reference.

Weight change patterns	No. of subjects	No. events	Model 1	Model 2	Model 3
			HR (95%CI)	HR (95%CI)	HR (95%CI)
Stable normal	10,379	334	Ref	Ref	Ref
Maximum overweight	10,215	356	1.00 (0.80, 1.26)	1.24 (0.99, 1.55)	**1.26 (1.01, 1.57)**
Obese to non-obese	255	14	1.81 (0.88, 3.70)	**2.15 (1.05, 4.42)**	1.95 (0.95, 4.00)
Non-obese to obese	5001	256	**1.48 (1.17, 1.87)**	**1.69 (1.33, 2.14)**	**1.70 (1.35, 2.15)**
Stable obese	1509	75	**1.47 (1.07, 2.02)**	**1.69 (1.22, 2.33)**	**1.66 (1.21, 2.29)**

All estimates accounted for complex survey designs and the analyses used the sample weights, stratification and clustering suggested by the CDC.

Significant results are in boldface type.

Model 1: Non-adjusted model.

Model 2: Adjusted for age at ten years before baseline, gender, race/ethnicity (Mexican American, non-Hispanic White, non-Hispanic Black, and other race).

Model 3: Adjusted for age at ten years before baseline, gender, race/ethnicity (Mexican American, non-Hispanic White, non-Hispanic Black, and other race), baseline education level (less than high school, high school or equivalent, college or above), baseline family income (family income-poverty ratio, 0–1.0, 1.1–3.0, > 3.0), baseline smoking status (ever smoke and never smoke).

**Table 3:** Hazard ratios (HRs) and 95% confidence intervals (CIs) of incident asthma with weight change patterns across adulthood in NHANES 2001–2018 used the stable obesity category as a reference.

Weight change patterns	No. of subjects	No. events	Mode 1	Model 2	Model 3
			HR (95% CI)	HR (95% CI)	HR (95% CI)
Stable obese	1509	75	Ref	Ref	Ref
Stable normal	10,379	334	**0.68 (0.49, 0.94)**	**0.59 (0.43, 0.82)**	**0.60 (0.44, 0.83)**
Maximum overweight	10,215	356	**0.68 (0.49, 0.96)**	0.73 (0.52, 1.04)	0.76 (0.54, 1.07)
Non-obese to obese	5001	256	1.23 (0.59, 2.57)	1.00 (0.71, 1.41)	1.02 (0.73, 1.44)
Obese to non-obese	255	14	1.01 (0.73, 1.40)	1.28 (0.61, 2.69)	1.18 (0.56, 2.49)

All estimates accounted for complex survey designs and the analyses used the sample weights, stratification and clustering suggested by the CDC.

Significant results are in boldface type.

Model 1: Non-adjusted model.

Model 2: Adjusted for age at 10 years before baseline, gender, race/ethnicity (Mexican American, non-Hispanic White, non-Hispanic Black, and other race).

Model 3: Adjusted for age at 10 years before baseline, gender, race/ethnicity (Mexican American, non-Hispanic White, non-Hispanic Black, and other race), baseline education level (less than high school, high school or equivalent, college or above), baseline family income (family income-poverty ratio, 0–1.0, 1.1–3.0, > 3.0), baseline smoking status (ever smoke and never smoke).

Since the maximum overweight was heterogeneous, we further analyzed this relationship in participants with overweight at age 25 or 10 years before baseline, which is presented in Table 4. The stable overweight group and weight change from overweight to obesity had higher risk of asthma (HR 1.48, 95% CI 1.07–2.04 and HR 1.47, 95% CI 1.03–2.08) compared with the stable normal group.

**Table 4:** Hazard ratios (HRs) and 95% confidence intervals (CIs) of incident asthma with participants with overweight at age 25 or 10 years before baseline in NHANES 2001–2018.

Weight change patterns	No. of subjects	No. of events	Mode 1	Model 2	Model 3
			HR (95%CI)	HR (95%CI)	HR (95%CI)
Stable normal	10,379	334	Ref	Ref	Ref
Normal to overweight	382	9	0.59 (0.26, 1.36)	0.72 (0.31, 1.66)	0.70 (0.30, 1.61)
Overweight to normal	6815	243	1.03 (0.81, 1.31)	1.18 (0.92, 1.50)	1.20 (0.94, 1.53)
Stable overweight	2842	97	**1.23 (1.02, 1.93)**	**1.46 (1.05, 2.02)**	**1.48 (1.07, 2.04)**
Overweight to obesity	2335	98	1.10 (0.78, 1.56)	**1.45 (1.02, 2.06)**	**1.47 (1.03, 2.08)**

All estimates accounted for complex survey designs and the analyses used the sample weights, stratification and clustering suggested by the CDC. Significant results are in boldface type.

Model 1: Non-adjusted model.

Model 2: Adjusted for age at 10 years before baseline, sex, race/ethnicity.

Model 3: Adjusted for age at 10 years before baseline, sex, race/ethnicity, baseline education level, baseline family income, baseline smoking status.

**Figure 2:**
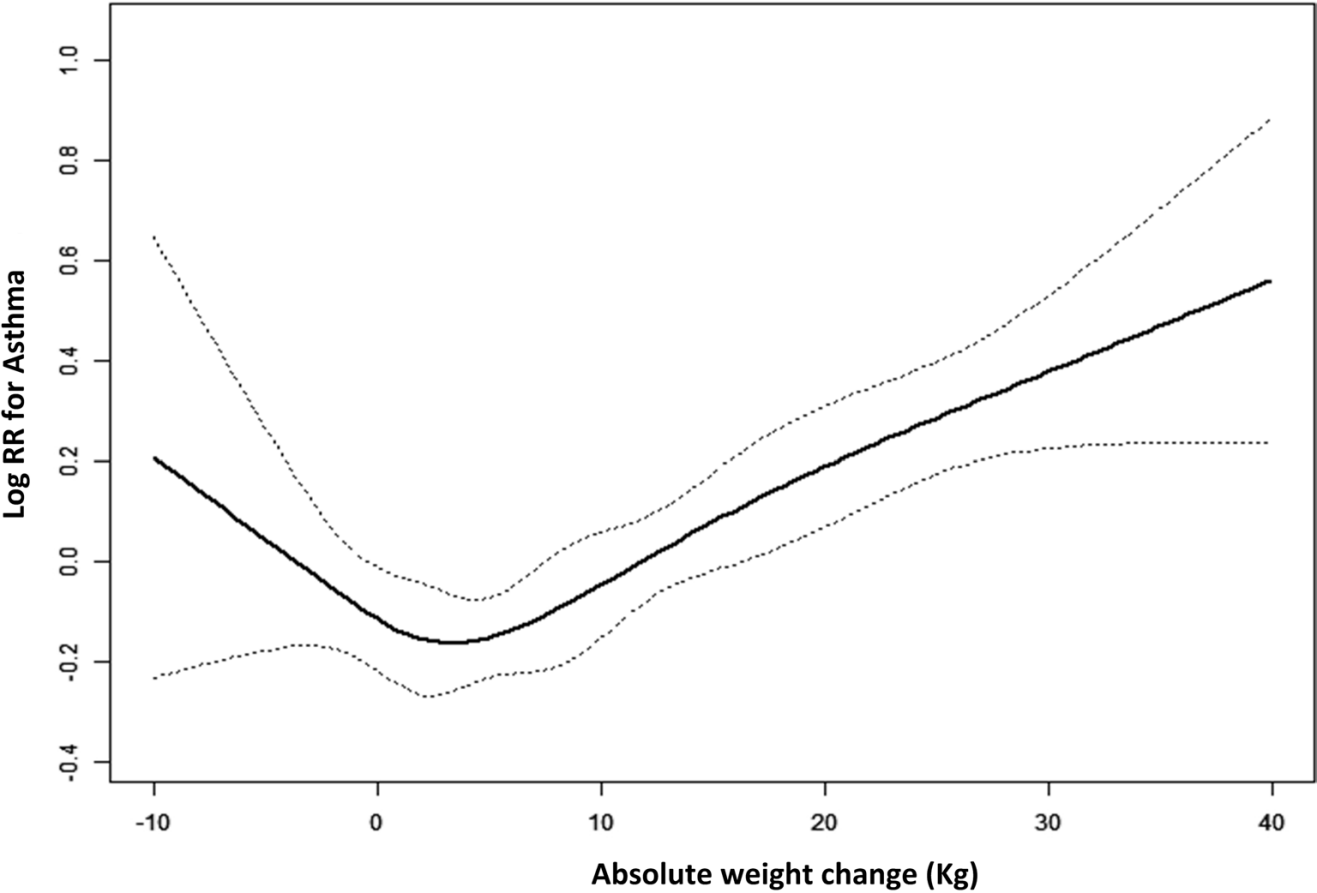
General additive models demonstrate the relationship between absolute weight change across adulthood and the risk of asthma. The resulting figures show the predicted log (relative risk) in the y-axis and the absolute weight change in the x-axis. The model was adjusted for gender, age at ten years before baseline, race/ethnicity, baseline family income, baseline education levels, and baseline smoking status.

The association between the absolute weight change and the risk of asthma is shown in Table 5 and Fig. 2. We also found a non-liner relationship between absolute weight change and the risk of asthma. As a continuous variable, a per 5 kg increment in weight change was associated with a 6% higher risk of asthma (HR 1.06, 95% CI 1.03, 1.09). As a categorical covariate, individuals with weight gain ≥ 20 kg had a higher risk of asthma (HR 1.45, 95% CI 1.11–1.89) compared with individuals with weight change within 2.5 kg. The adjusted cumulative hazard curves of asthma for each absolute weight change group are presented in Supplementary Fig. S4. The cumulative incidence of weight gain ≥ 20 kg was the highest obtained.

**Table 5:** Hazard ratios (HRs) and 95% confidence intervals (CIs) of incident asthma with absolute weight change patterns across adulthood.

Weight change patterns	No. of subjects	No. events	Model 1	Model 2	Model 3
			HR (95% CI)	HR (95% CI)	HR (95% CI)
Continuous (per 5 kg increment)	27,359	1035	**1.06 (1.03, 1.09)**	**1.05 (1.02, 1.09)**	**1.06 (1.03, 1.09)**
**Category**					
Weight loss > 2.5	1599	61	1.05 (0.69, 1.59)	1.07 (0.70, 1.63)	0.96 (0.68, 1.59)
Weight change within 2.5 kg	6245	222	Ref	Ref	Ref
2.5 kg ≤ Weight gain < 10 kg	8390	262	0.86 (0.65, 1.15)	0.87 (0.65, 1.15)	0.91 (0.68, 1.21)
10 kg ≤ Weight gain < 20 kg	6412	243	1.18 (0.91, 1.53)	1.19 (0.91, 1.55)	1.25 (0.95, 1.64)
Weight gain ≥ 20 kg	4713	247	**1.41 (1.07, 1.85)**	**1.40 (1,07, 1.84)**	**1.45 (1.11, 1.89)**

All estimates accounted for complex survey designs and the analyses used the sample weights, stratification and clustering suggested by the CDC.

Significant results are in boldface type.

Model 1: Non-adjusted model.

Model 2: Adjusted for age at 10 years before baseline, gender, race/ethnicity (Mexican American, non-Hispanic White, non-Hispanic Black, and other race).

Model 3: Adjusted for age at 10 years before baseline, gender, race/ethnicity (Mexican American, non-Hispanic White, non-Hispanic Black, and other race), baseline education level (less than high school, high school or equivalent, college or above), baseline family income (family income-poverty ratio, 0–1.0, 1.1–3.0, > 3.0), baseline smoking status (ever smoke and never smoke).

### Stratified analyses and sensitivity analysis

The results of the stratified analyses according to baseline age, gender and smoking strata are shown in Table 6. In each subgroup, non-obese to obese group was associated with a higher risk of asthma. Stable obesity significantly increased the risk of asthma in the female subgroup, baseline age < 50 years old subgroup and non-smoking subgroup. The results of interaction analyses indicated that age, gender, and smoking status did not significantly affect these associations. (P-value for interaction > 0.05).

**Table 6:** Hazard ratios (HRs) and 95% confidence intervals (CIs) of incident asthma with weight change patterns across adulthood stratified by baseline age, gender and smoking status.

	Number of events	Weight change patterns, adjusted-HR (95%CI)					P value for interaction
		Stable normal	Maximum overweight	Obese to non-obese	Non-obese to obese	Stable obese	
**Age**							0.7310
< 50	510	Ref	**1.41 (1.08 1.85)**	2.11 (0.89, 4.97)	**1.73 (1.25, 2.39)**	**1.71 (1.17, 2.49)**	
≥ 50	525	Ref	1.06 (0.78, 1.43)	1.58 (0.47, 5.30)	**1.61 (1.16, 2.22)**	1.63 (0.86, 3.07)	
**Gender**							0.7566
Male	358	Ref	1.44 (0.97, 2.13)	2.96 (0.93, 9.41)	**1.66 (1.09, 2.53)**	1.46 (0.71, 3.00)	
Female	677	Ref	1.20 (0.92 1.58)	1.48 (0.66, 3.34)	**1.75 (1.34, 2.30)**	**1.79 (1.27, 2.52)**	
**Smoking status**							0.2156
Ever smoking	570	Ref	1.25 (0.96, 1.63)	1.15 (0.43, 3.09)	**1.76 (1.30, 2.39)**	1.31 (0.81, 2.14)	
Never smoking	464	Ref	1.29 (0.93, 1.79)	**3.61 (1.32, 9.82)**	**1.66 (1.21, 2.30)**	**2.12 (1.34, 3.35)**	

All estimates accounted for complex survey designs and the analyses used the sample weights, stratification and clustering suggested by the CDC.

Risk estimates were adjusted for gender (not adjusted in stratified analysis by gender), age at 10 years before baseline (not adjusted in stratified analysis by age), race/ethnicity, baseline family income, baseline education, and baseline smoking status (not adjusted in stratified analysis by smoking status).

Significant results are in boldface type.

We further performed sensitivity analyses which are shown in Supplementary Table S4. After excluding the missing values for covariates, participants in non-obese to obese and stable obese groups had higher risk of asthma after adjustment of all cofounders (HR 1.73, 95% CI 1.35–2.21 and HR 1.70, 95% CI 1.21–2.39, respectively). Except for participants who were underweight at age 25 or 10 years ago before baseline, the results suggest that changing from non-obese to obese, and stable obese was also associated with a higher risk of asthma (HR 1.71, 95% CI 1.34–2.18 and HR 1.65, 95% CI 1.19–2.28, respectively), which is consistent with the results in Table 2.

We separately investigated the relationship between weight change in the underweight group at age 25 and the risk of asthma. There were 1374 underweight participants at 25 years old, accounting for 5% of the total participants. Two hundred twenty of them maintained underweight at middle age (including nine cases of asthma), 825 (including 27 cases of asthma) of them became normal weight, 227 (6 cases of asthma) of them became overweight, and 102 of them (5 cases of asthma) became obese. Further, there was no significant increase in the risk of asthma in the underweight group compared with those who maintained normal weight (Supplementary Table S5).

## Discussion

In this study we used data from nine waves of the NHANES survey to investigate the relationship between weight change patterns and the risk of asthma. The results suggest weight change patterns across adulthood are significantly associated with the risk of asthma, with participants who enter young adulthood with a normal BMI and continue to maintain a normal BMI during middle adulthood having the lowest risk of asthma. Furthermore, participants who are stable overweight, stable obese and those who gain weight from non-obese to obese are associated with an increased risk of asthma.

Numerous studies have found adults with overweight and obesity are associated with a higher risk of asthma than adults with normal weight^16–21^**.** Recently, results from two studies (using Norwegian and European populations) suggest a causal relationship between BMI and the risk of asthma in adults by using bi-directional Mendelian randomized analysis, with the risk of asthma significantly increased with the increase in BMI^22, 23^. These results are consistent with our findings. In this study, we found that overweight or obese participants in age 25 or middle age (age 10 years before baseline) had a significantly increased risk of asthma in later life.

Another finding of this study is that weight changes are common across adulthood. Only a few previous studies have examined the relationship between weight change and the risk of asthma in adults, and the results are controversial. The Nurses' Health Study II (1596 cases/85,911 participants, using a four-year follow-up) and the E3N Cohort Study (372 cases /67,299 participants, using a 3-year follow-up) found weight gain significantly increased the risk of developing asthma^24, 25^, which are consistent with our findings. While other studies are inconsistent with our results, the Zurich Cohort Study which included 591 participants with 20-years cumulative asthma incidences (8.1%) and the Finnish Twin Cohort which included 10,597 participants with 9-years cumulative asthma incidences (1.2%) found no association with weight gain or obesity and later asthma in both men and women^17, 26^. Reasons for those inconsistent results may include differences in baseline age, differences in sample size and differences in the definition of weight change. In sum, most of those studies focused on a specific population and some included only women, so the results may not be generalizable to whole populations. Our study used nationally representative data, which complemented the previous studies, and the results also confirm that weight gain across adulthood presents higher risk for asthma.

Two previous articles have reported the association between weight change patterns and asthma in adults. Kim et al. used the KNHANES data with 29,936 participants who developed asthma after a 3-year follow-up in 245,593 adults and found that weight gain increased the risk of asthma compared with maintaining a normal weight, again consistent with our results. They also reported that no association was found between weight gain from normal or underweight to obese and the risk of asthma^14^, which appears inconsistent with our results. The inconsistency might be due to two reasons. First, the two studies observed weight change over different periods of time. Our study assessed weight change across adulthood with an average period of 25 years, whereas their study assessed weight change for only three years between 2005 and 2008. Secondly, they used different reference groups, included different number of cases and there were also some differences in the definitions of weight change patterns^14^. Wang et al. found the risk of developing asthma increased 1.61 and 1.43 in the non-obese to obese group and the stable obese group, respectively by using the data from NHANES (1999–2016)^15^. Their study focused on obese participants. However, there were more overweight participants than obese ones. Of the 27,359 participants, 20.75% were overweight at age 25, rising to 36.66% at 10 years before baseline. There were 10,031 overweight participants and 6510 obese participants (36.66% versus 27.40%) at 10 years before the survey (middle adulthood). We further analyzed the association between weight change and the risk of asthma in overweight participants. The incidences of asthma are also significantly higher among these participants who were stable overweight and changed from overweight to obese in early adulthood. Therefore, we emphasize that weight changes not only in obese participants, but also in overweight participants are significantly associated with the risk of asthma, which complemented the previous study.

This study found no association between absolute weight loss and incident asthma, which is consistent with previous results^24, 25, 27^. This study also found weight loss from obese to non-obese was not significantly associated with the risk of asthma, possibly due to a relatively small sample size as only 0.9% participants lost weight from obesity to normal. Thus, we should interpret these results with caution.

Obesity and excess fat accumulation in the body may promote the development and progression of asthma through three pathways, including inflammatory, metabolic, and mechanistic pathways. Animal research suggested that pro-inflammatory factors increased in bronchoalveolar lavage fluid of high-fat diet-ovalbumin mice (HFD-OVA) compared with lean mice^7^. Other research found that obesity alters the lung function of participants by reducing the movement of the diaphragm to increase airway restriction^28^. In sum, in the inflammatory and metabolic pathways, adipose tissue promotes airway obstruction and affects lung function by secreting excessive pro-inflammatory factors IL-6, IL-1β, TNFα, and TGFβ, while the mechanical pathways affect lung function by increasing airway restriction that promotes asthma^7, 29^.

In this study, we used a large and nationally representative sample, analyzed weight status at each time point, weight change patterns, absolute weight change, and the risk of asthma. In addition, we analyzed the association between weight change patterns and the risk of asthma in underweight, normal weight, overweight, and obese participants in detail. These are the strengths of this study. Also, considering that some potential factors (smoking, family income and education) could affect the results, we used different models to analysis the relationship and further adjusted these confounding factors on the basis of model 2. The result of the analysis showed that compared with the unadjusted model, the associations between body weight change and the risk of asthma did not change in the adjusted models.

However, there are some limitations in the current study. First, the self-reported weight rather than measured weight was used in this study. Though a high degree of consistency between self-reported weight and measured weight has been demonstrated in previous articles, some misclassification has likely occurred^30, 31^. And we used self-reported scale instead of lung function to identify participants with asthma, which may also lead to misclassification. Second, the sample size of weight loss from youth to middle age was small, which may limit the precision of our estimates; Third, we could not adjust for some confounding variables including dietary factors, alcohol consumption, and physical activity because these data at 10 years before baseline were not collected. Lastly, we did not analyze the association between the change in other obesity related markers, such as fat mass and waist circumference and incident asthma, because that data was not collected at the two-time point intervals.

## Conclusions

In conclusion, our investigation indicates that in a sample of U.S. adults, stable obesity, adult with overweight and weight gain from normal or overweight to obese during adulthood are associated with increased risks of asthma. Maintaining normal weight during adulthood had the lowest risk of asthma. We found no significant association between weight loss and asthma. This may be due to small sample size, so more studies are needed to assess this association. In addition, we emphasized that weight changes not only in obese participants but also in overweight participants are associated with the risk of asthma, and weight management in these participants should be emphasized.

## Material and methods

### Data source

All data used in this study for analysis were obtained from the NHANES (2001–2018), which was conducted by the National Center for Health Statistics of the Centers for Disease Control and Prevention (CDC). NHANES is a series of ongoing cross-sectional surveys and used a stratified multistage probability sample design to collect health-related data of nationally representative samples of the US population^32–35^.

All participants signed the informed consent forms during the period of recruitment, and all protocols were approved by the research ethics review board of the National Center for Health Statistics of CDC. We confirmed that all methods were performed in accordance with relevant guidelines and regulations.

### Study design and population

We collected data on adults aged 40 or over at the time of the survey from the nine cycles of the consecutive NHANES 2001–2018. We defined the time at which participants had a physical examination as the baseline, and weight change was estimated from participants' self-reported weight at age 25 (weight_age25_) and weight at 10 years before baseline (weight_10prior_). We defined incident asthma from a self-administered questionnaire which was completed at the clinic visit. We used the reported age at diagnosis to establish the time of incident asthma. The present study design is shown in Figure S1 and the design of NHANES has been shown in previous studies^36–38^.

Referring to several recent studies, the exclusion criteria were as follows^36, 37^: participants who were younger than 40 years old; participants who did not have asthma diagnostic information and diagnosis time of asthma; participants who did not have data on BMI at age 25 (BMI_age25_) and BMI 10 years before baseline (BMI_10prior_); and participants whose asthma onset date was before the initiation of a 10-year follow-up. Finally, we enrolled 27,359 participants in the current study.

### Weight change measures and covariates

Weight_age25_, weight_10prior_ and height at age 25 were recalled when participants were interviewed. Measured height at physical examination is used to calculate BMI_10prior_. When the participant was < 50 years old, it was also used to calculate BMI_age25_. But when the participant was ≥ 50 years of age, height at age 25 was used to calculate BMI_age25_.

Referring to several recent studies^36, 37^, according to BMI_age25_ and BMI_10prior_, we defined five patterns of weight change which are shown in Supplementary Table S1, including stable normal (both BMI < 25), non-obese to obese (BMI_age25_ < 30 and BMI_10prior_ ≥ 30), obese to non-obese (BMI_age25_ ≥ 30 and BMI_10prior_ < 30), maximum overweight (25 ≤ BMI ≤ 29.9 at age 25 or ten years before baseline, but BMI not ≥ 30 at the other time), and stable obese (both BMI ≥ 30). Age _10 prior_ ranged from 30 to 75 years old. Those whose weight change patterns were from age 25 to age 30 accounted for 2.7% of the total participants. To compare with previous studies^14, 25^, we also further analyzed the association between absolute weight change during this period and incident asthma. We calculated the absolute weight change by subtracting weight_age25_ from weight_10prior._ Then, we divided the absolute weight changes into five groups: weight loss > 2.5 kg, weight change within 2.5 kg (the absolute weight loss or weight gain ≤ 2.5 kg), 2.5 kg < weight gain ≤ 9.9 kg, 10 kg ≤ weight gain ≤ 19.9 kg, and weight gain ≥ 20.0 kg, weight change within 2.5 kg was used as the reference group. We chose these categories for comparison with the previous studies^15, 36–38^.

Covariate data were obtained from demographic and questionnaire surveys, including gender, baseline age, ethnicity (Mexican American, non-Hispanic White, non-Hispanic Black, and other race), baseline smoking status, baseline education level (less than high school, high school or equivalent, college or above) and baseline family income. Poverty income ratio (0–1.0, 1.1–3.0, > 3.0) was used to reflect family income. Smoking status included never smoking and ever smoke defined as “smoked at least 100 cigarettes in lifetime”.

### Statistical analysis

According to NHANES analytic guidelines, we analyzed the data using a weight suggested by the CDC to account for the complex survey design (including oversampling). The categorical variables are presented as percentages and the continuous variables are expressed as means ± SD or medians [interquartile range (IQR)]. All the variables were compared among different groups by the weighted chi-square tests when they were categorical variables, and by the weighted linear regression models when they were continuous variables. We assessed BMI at each time point, the weight change pattern and the absolute weight change during adulthood and risk of asthma respectively.

First, we used Cox Proportional Hazards Regression Analyses to evaluate the association of BMI at each time point (BMI_age25_ and BMI_10prior_) and the risk of asthma. We divided the participants into six groups: underweight, normal weight, overweight, class 1 obesity, class 2 obesity, class 3 obesity and we used normal weight as a reference.

Second, we evaluated the association of weight change patterns and incident asthma. To assess the increased risk of incident asthma, the stable normal category was used as a reference to compare with all other categories. On the contrary, the stable obesity category was used as a reference to compare with all other categories to assess the decreased risk. In this study, we used three sequential models to control for potential confounders. Model 1 is a non-adjusted model. Model 2 was adjusted for age, gender and race. Model 3 was adjusted for age, gender, race, education, family income and smoking status. Because 2298 participants missed information regarding family income, which was more than 10% of total participants, we set these mission values as another level of the categorical covariate to be adjusted in Model 2. Twenty-three and 15 participants missed the data of baseline education levels and smoking status which represented less than 1% of total participants. We continued our analysis without making any further adjustments. We used the survival curves of weight change patterns to test the proportional hazards assumption and no significant deviation from proportionality in hazards over time was detected. Then, we performed the subgroup analysis stratified by baseline age, gender, and smoking status to determine whether there were differences among each subgroup. We divided the participants into two groups by baseline age at 50 years old. We also performed the interaction tests between age, gender, smoking status and weight change patterns for the risk of asthma. Gender, smoking status and weight change patterns were treated as nominal variables. Age group was treated as an ordinal variable and the median of each category was used for analysis. The adjusted log likelihood ratio test was used to analyze the interaction.

Third, we evaluated the association of absolute weight change and incident asthma adjusted for the same covariates. Firstly, we performed the analysis by taking absolute weight change as a continuous variable. Secondly, we took the absolute weight change as a categorical covariate and compared with all other categories. We used body weight change within the 2.5 kg category as a reference. Furthermore, we used generalized linear models with a logit link to evaluate the nonlinearity between them.

The Cox Proportional Hazards Regression Analyses was used to conduct the following sensitivity analyses. First, we excluded participants with missing variables. Second, to reduce the effect of underweight, we excluded underweight participants. Third, considering the heterogeneity of the maximum overweight group, which includes participants with weight change from normal to overweight, from overweight to normal, and stable overweight, we divide those participants into three groups according to BMI_age25_ and BMI_10prior._ Finally, we analyzed the association between the weight change in underweight group at age 25 and the risk of asthma.

We used the EmpowerStats software (www.empowerstats.com version R.3.4.3) and statistical software package R to process and analyze all our data. A two-tailed P value < 0.05 was considered statistically significant.

## Supplementary Information

Supplementary Information.

## Data Availability

The data are available on the National Health and Nutrition Examination Survey website (https://www.cdc.gov/nchs/nhanes/).
