# Supplementary material for: Association of weight change patterns across adulthood with incident asthma: a retrospective cohort study
Source: Sci Rep. 2022 Jun 13;12:9756. doi: 10.1038/s41598-022-13555-w (PMC9192600; doi:10.1038/s41598-022-13555-w)
Supplement: Supplementary file 1 — Supplementary Information. [file 41598_2022_13555_MOESM1_ESM.pdf]

Association of weight change patterns across adulthood with incident asthma: a retrospective cohort study

Wei Zhang, MD1*, Jie Du, MD[2], Shaochun Wang, MD[1], Huihui Ma, MD[1]

Table S1. The definition of weight change patterns

Weight change patterns	BMI at age 25	BMI at 10 years before baseline
Stable normal	<25 kg/m^2	<25 kg/m^2
Maximum overweight	≥25 kg/m^2，<30 kg/m^2	<30 kg/m^2
	<30 kg/m^2	≥25 kg/m^2，<30 kg/m^2
Non-obese to obese	<30 kg/m^2	≥30 kg/m^2
Obese to non-obese	≥30 kg/m^2	<30 kg/m^2
Stable obese	≥30 kg/m^2	≥30 kg/m^2

Table S2. Hazard ratios (95% confidence intervals) of incident asthma with BMI status at age 25 in the NHANES 2001-2018

BMI status at age 25	Number of subjects	Number of events	Model 1	Model 2	Model 3
			HR (95% CI)	HR (95% CI)	HR (95% CI)
Underweight	1374	47	1.11(0.72, 1.70)	1.03(0.63, 1.50)	0.99(0.64, 1.52)
Normal	18544	691	Ref	Ref	Ref
Overweight	5677	208	0.90(0.72, 1.14)	1.20(0.95, 1.52)	1.26(0.99, 1.59)
Obesity	1764	54	1.20(0.82, 1.7)	1.41(0.97, 2.06)	1.40(0.94, 2.07)

Class 2 Obesity	**306**	**20**	**1.94(1.04, 3.61)**	**1.99(1.09, 3.64)**	**1.93(1.04, 3.57)**
Class 3 Obesity	219	15	1.45(0.72, 2.91)	1.32(0.67, 2.62)	1.32(0.65, 2.72)

All estimates accounted for complex survey designs and all analyses used the sample weights, stratification and clustering suggested by the CDC

Significant results are in boldface type.

BMI variables were categorized into six groups: underweight (<18.0) and normal weight (18.0-24.9), overweight (25.0-29.9), Class 1 obesity (30.0-34.9), Class 2 obesity (35.0-39.9), and Class 3 obesity (≥40).

Model 1: Non adjusted model

Model 2: adjusted for sex, race/ethnicity

Model 3: adjusted for sex, race/ethnicity, baseline education level, baseline family income-poverty ratio level, baseline smoking status

Table S3. Hazard ratios (95% confidence intervals) of incident asthma with BMI status at 10 years before baseline in the NHANES 2001-2018

BMI status at 10 years before baseline	Number of subjects	Number of events	Model 1	Model 2	Model 3
			HR (95% CI)	HR (95% CI)	HR (95% CI)
Underweight	331	12	1.47(0.64, 3.37)	1.29(0.56, 2.98)	1.16(0.51, 2.65)
Normal	10487	335	Ref	Ref	Ref
Overweight	10031	357	1.05(0.84, 1.32)	**1.29(1.03, 1.62)**	**1.31(1.04, 1.64)**
Class 1 Obesity	4232	189	**1.37(1.08, 1.75)**	**1.63(1.28, 2.08)**	**1.63(1.28, 2.08)**
Class 2 Obesity	1346	80	**1.92(1.34, 2.74)**	**2.12(1.49, 3.03)**	**2.15(1.51, 3.05)**
Class 3 Obesity	932	62	**1.53(1.07, 2.20)**	**1.46(1.02, 2.09)**	**1.44(1.01, 2.06)**

All estimates accounted for complex survey designs and all analyses used the sample weights, stratification and clustering suggested by the CDC

Significant results are in boldface type.

BMI variables were categorized into six groups: underweight (<18.0), normal weight (18.0-24.9), overweight (25.0-29.9), Class 1 obesity (30.0-

34.9), Class 2 obesity (35.0-39.9), and Class 3 obesity (≥40).

Model 1: Non-adjusted model

Model 2: Adjusted for age at ten years before baseline, sex, race/ethnicity

Model 3: Adjusted for baseline age at ten years before baseline, sex, race/ethnicity, baseline education level, baseline family income, baseline smoking status

Table S4. Sensitivity analyses of the associations between weight change patterns and incident asthma across adulthood in NHANES 2001-2018

Weight change patterns	No. of subjects	No. of events	Model 1	Model 1	Model 2
			HR (95%CI)	HR (95%CI)	HR (95%CI)
Sensitivity analysis 1					
Stable normal	9489	306	Ref	Ref	Ref
Maximum overweight	9357	334	1.02(0.81, 1.30)	1.25(0.99, 1.58)	**1.29(1.02, 1.63)**
Obese to non-obese	241	13	1.73(0.82, 3.65)	1.99(0.93, 4.26)	1.85(0.87, 3.90)
Non-obese to obese	4567	237	**1.53(1.20, 1.95)**	**1.70(1.33, 2.17)**	**1.73(1.35, 2.21)**
Stable obese	1379	68	**1.52(1.08, 2.13)**	**1.68(1.21, 2.33)**	**1.70(1.21, 2.39)**
Sensitivity analysis 2					
Stable normal	9235	296	Ref	Ref	Ref
Maximum overweight	9977	350	1.02(0.81, 1.29)	1.25(0.99, 1.58)	**1.26(1.00, 1.59)**
Obese to non-obese	254	13	1.69(0.79 3.63)	1.99(0.93, 4.26)	1.82(0.85, 3.91)
Non-obese to obese	4899	251	**1.51(1.19, 1.93)**	**1.70(1.33, 2.17)**	**1.71(1.34, 2.18)**
Stable obese	1509	75	**1.50(1.09, 2.07)**	**1.68(1.21, 2.33)**	**1.65(1.19, 2.28)**

Significant results are in boldface type.
Sensitivity analysis 1: A total of 2326 participants with any missing values for covariates were excluded.
Sensitivity analysis 2： A total of 1485 underweight participants were excluded due to being underweight at either time point during the weight change intervals.
Model 1: Non-adjusted model
Model 2: adjusted for age at ten years before baseline, sex, race/ethnicity
Model 3: adjusted for age at ten years before baseline, sex, race/ethnicity, baseline education level, baseline family income, baseline smoking status

Table S5. Hazard ratios (HRs) and 95% confidence intervals (CIs) of incident asthma with participants in the underweight group at age 25

Weight change patterns	No. of subjects	No. events	HR (95%CI)
Stable normal	9235	296	Ref
Stable underweight	220	9	1.54(0.56, 4.19)
Underweight to normal weight	825	27	1.07(0.59, 4.19)
Underweight to overweight	227	6	1.17(0.44, 3.09)
Underweight to obesity	102	5	1.32(0.49, 3.53)

Adjusted for gender, age at ten years before baseline, race, baseline family income, baseline education levels, baseline smoking status

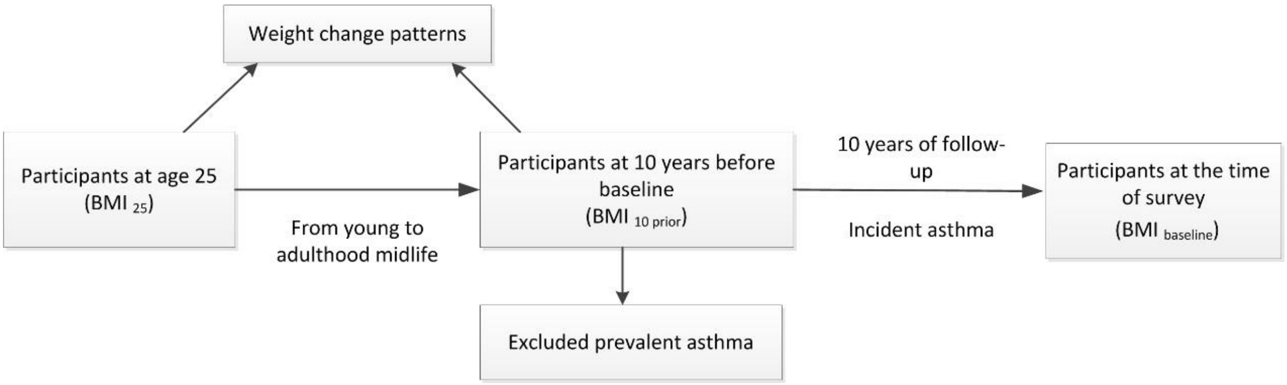

Figure S1 Survival analysis study design: weight change and asthma onset

A

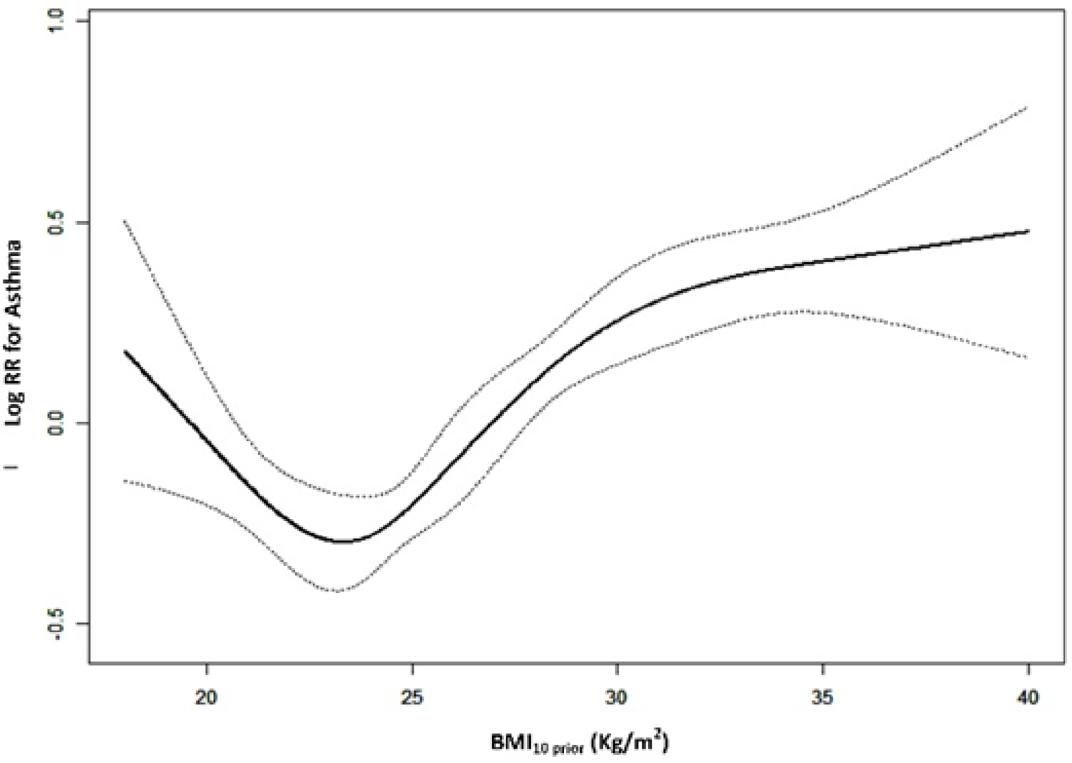

B

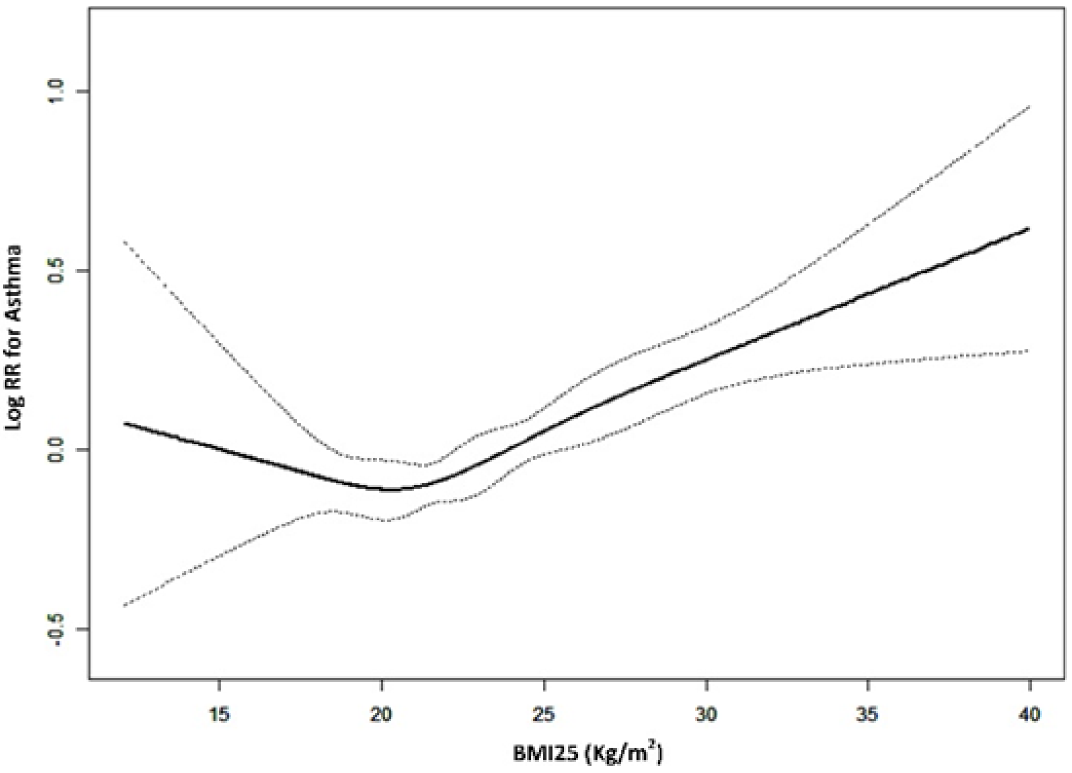

Figure S2 General additive models demonstrate the relationship between BMI at

each time point and the risk of asthma.

(A): Association between BMI $_{10prior}$ and the risk of asthma

(B): Association between BMI $_{age25}$ and the risk of asthma

The resulting figures show the predicted log (relative risk) in the y-axis and the absolute weight change in the x-axis. The model was adjusted for sex, age at ten years before baseline, race/ethnicity, family income, education levels, smoking status.

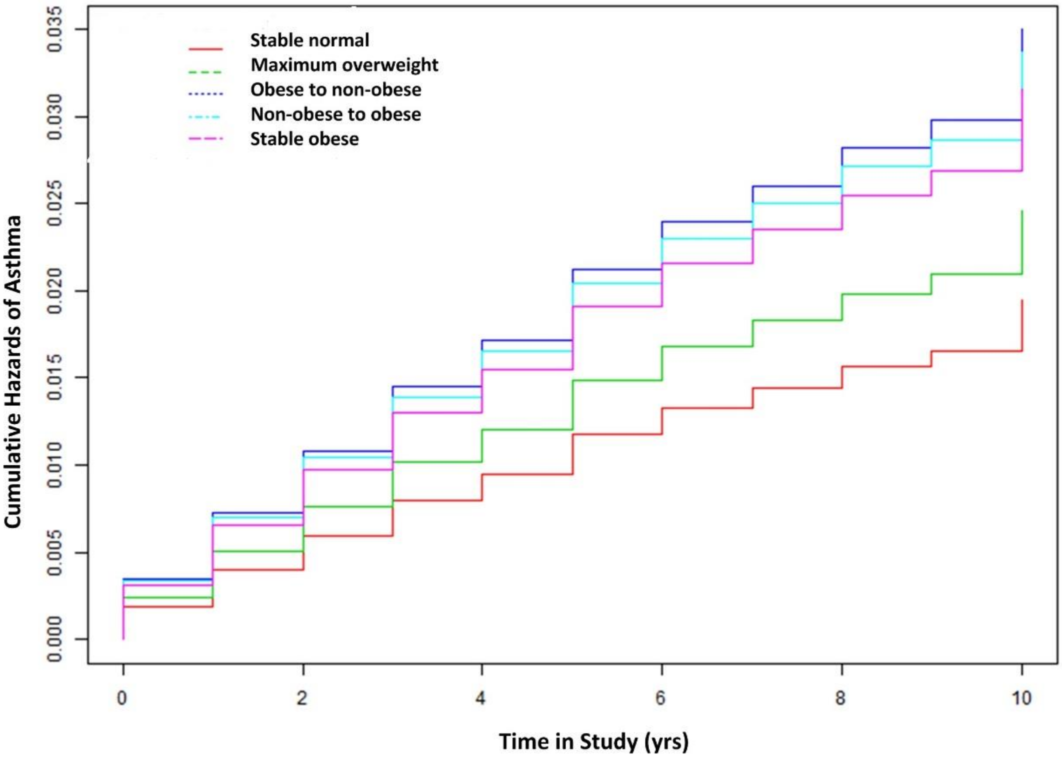

Figure S3 Adjusted cumulative hazard curves of the risk of asthma in later life for weight change patterns during adulthood

Multivariable Cox regression model adjusted for sex, age at ten years before baseline, race/ethnicity, family income, education levels, smoking status.

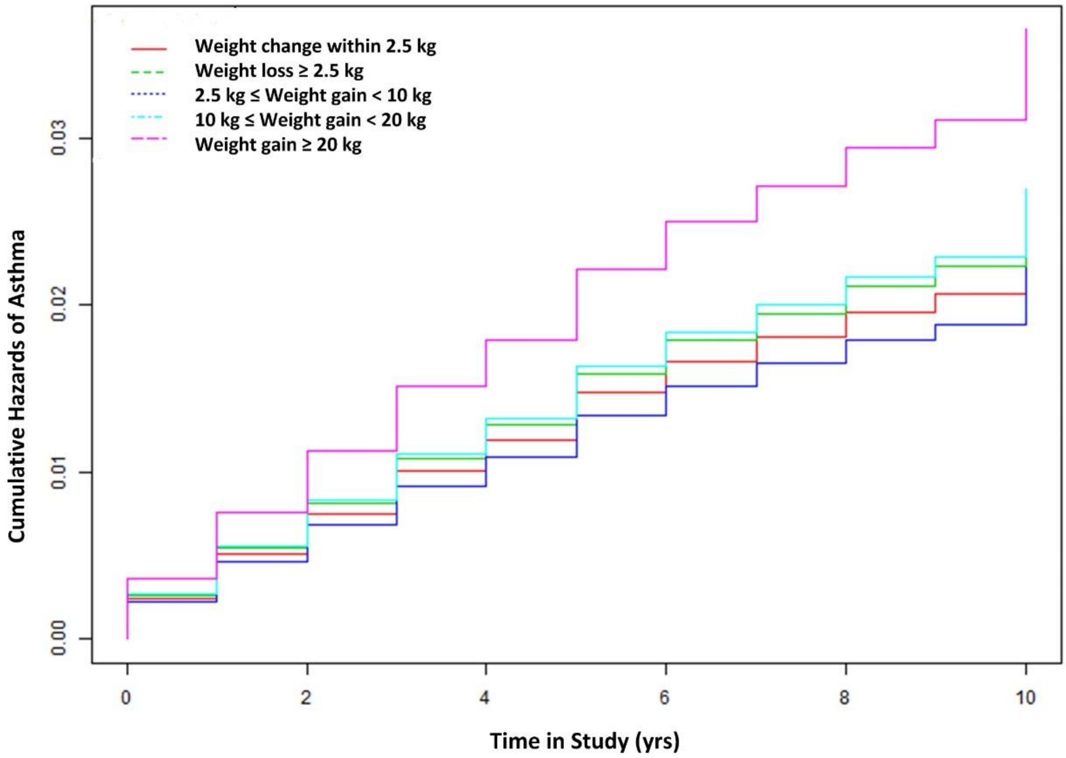

Figure S4 Adjusted cumulative hazard curves of the risk of asthma in later life for absolute weight change category during adulthood

Multivariable Cox regression model adjusted for sex, age at ten years before baseline, race/ethnicity, family income, education levels, smoking status.
